# A Case Report on a Young Male Taking Methylphenidate With Fasciculations and Dysphagia: Correlation or Causation?

**DOI:** 10.7759/cureus.32114

**Published:** 2022-12-01

**Authors:** António Paiva, Adriana Pascoal, Carolina Lourenço, César Pires, Jorge Lains

**Affiliations:** 1 Physical Medicine and Rehabilitation, Centro de Medicina de Reabilitação da Região Centro - Rovisco Pais, Tocha, PRT

**Keywords:** methylphenidate hydrochloride, iatrogenic effect, fasciculation, oropharyngeal dysphagia, motor neuron disease

## Abstract

Concurrent fasciculations and oropharyngeal dysphagia (OD) can be presenting signs of motor neuron disease (MND); however, there are other causes for OD (neoplasms, surgery, and gastroesophageal diseases, among others). Fasciculations (anxiety, benign, or iatrogenic) are an uncommon side effect (<1%) of methylphenidate. A 30-year-old male noticed fasciculations in both gastrocnemii, reporting gradual cranial progression, culminating in diffuse fasciculations with facial involvement. One month later, he reported OD for solids and occasional cough for liquids. He denied weakness, fatigue, or weight loss. He has no relevant personal history, apart from attention deficit hyperactivity disorder diagnosed a year before and since then medicated with methylphenidate 40 mg id. He had no abnormal findings on neurological examination. Electromyography (EMG) and sinus CT were normal. Upper gastrointestinal (GI) endoscopy (EGD) showed reflux esophagitis grade C, which could explain OD, and he started esomeprazole 40 mg id. As there were no findings on EMG, an iatrogenic etiology for fasciculations was considered. He suspended methylphenidate for a month and, two months later, reported a substantial improvement in fasciculations and resolution of the OD with the introduction of esomeprazole.

Two simultaneous symptoms do not mean they are related. In this specific case, OD was the first symptom of gastroesophageal reflux disease (GERD), and fasciculations happened as a side effect of methylphenidate. This must be taken into consideration, as it can represent a confounding factor making the differential diagnosis more difficult. To the best of our knowledge, there are no published articles similar to this case report.

## Introduction

Fasciculations can be defined as spontaneous, fine, and intermittent contractions of muscle fibers [1]. Benign fasciculations are common and appear in 70% of healthy individuals at some point in their lives [2]. However, it is a frequent cause of medical consultation [3] given its relationship with amyotrophic lateral sclerosis (ALS), a disease that leads to the depletion of motor neurons in the anterior horn of the spinal cord and the pyramidal tract [1,3]. Several causes of benign fasciculations have been identified, the most common of which are stress, anxiety, caffeine, nicotine, and sleep deprivation [1,2].

Oropharyngeal dysphagia (OD) consists of difficulty or inefficiency in transporting the bolus from the mouth to the esophagus, through the pharynx [4]. Although there are several causes for OD (head and neck neoplasms, radiotherapy, surgery, gastroesophageal diseases, and aging, among others), neurological diseases (stroke, Parkinson’s disease, and motor neuron disease (MND)) are the most frequently associated [4]. It is estimated that OD affects >30% of stroke patients, and in Europe, around 40 million people suffer from OD [4]. Namely, in MND, dysphagia is present in 35%-80% of patients [5], an initial symptom in one-third of patients with ALS [6].

With this work, we intend to present the clinical case of a patient who resorted to a consultation of Physical Medicine and Rehabilitation (PMR) due to generalized fasciculations and OD.

This case was previously presented as a poster at the 16th International Society of Physical and Rehabilitation Medicine World Congress, Lisboa, Portugal, on July 3-7, 2022.

## Case presentation

We describe the case of a 30-year-old male who, in November 2019, noticed fasciculations in both gastrocnemii (Video 1), reporting gradual cranial progression, culminating in diffuse fasciculations with facial involvement in February 2020. Concomitantly, in March, he reported dysphagia for solids (described as food sticking at the base of the throat) and occasional cough for liquids. He denied any history of muscle wasting, muscle weakness, weight loss, or recurring falls. He denied smoking, caffeine abuse (drank 2-3 coffee during work days since he was 18), or sleep deprivation. However, he did report considerable anxiety three months prior to consultation that considerably exacerbated in March, which prompted him to seek medical attention in April 2020. He has no relevant personal history, except for a recent diagnosis of adult attention deficit (AAD), for which he started methylphenidate 40 mg a year before.

**Video 1:** Fasciculations The video was made by the patient and was reproduced in the first consultation.

Upon observation, we observed fasciculations in both calves. However, there was no evident amyotrophy, asymmetry, or gait claudication. Cranial nerve evaluation (including gag reflex), muscle strength (by medical research council), superficial and deep sensory functions, and deep tendon reflexes were normal upon evaluation.

Electromyography (EMG) and sinus CT revealed no pathological findings. Upper gastrointestinal (GI) endoscopy (EGD) was performed and showed reflux esophagitis grade C (Los Angeles classification). For this reason, he started esomeprazole 40 mg id.

He was again observed in the next month, with resolution of dysphagia, maintaining, however, widespread fasciculations. Since there were no abnormal findings on EMG and his anxiety levels had decreased substantially, an iatrogenic etiology for fasciculations was considered, namely, methylphenidate. He suspended the medication for a month and, two months later, reported a substantial improvement in fasciculations and improvement of the dysphagia after starting esomeprazole.

On follow-up, although he had a near-complete resolution of dysphagia and fasciculations, he was noticing the lack of methylphenidate, especially in a professional setting. With his psychiatrist’s supervision and agreement, we reintroduced the medication with a relapse of lower limb fasciculations two weeks later. We reduced the dosage to 30 mg with a reduction of the fasciculations and an acceptable work performance.

On his last evaluation, five months later, he had no constant fasciculations, reporting aggravation on days when he sleeps for fewer hours or when he drinks more coffee (usually above three per day).

## Discussion

This case presented a challenge for us to diagnose because of the unusual presentation. To this end, we thought it best to approach and summarize three points: the presenting symptoms and their interpretation, the differential diagnosis, and the definite diagnosis.

Symptom interpretation

Fasciculations

It is important to note that it is exceptionally rare that fasciculations alone are the presenting symptom of ALS [7]. Also, in benign fasciculation syndrome (BFS), although fasciculations can persist in a specific muscle over a long period of time, it usually has an intermittent behavior. In addition, there have been case reports of patients with cramps and fasciculations with no EMG abnormalities that persisted for one year that later developed progressive weakness, muscle wasting, and EMG abnormalities, being ultimately diagnosed with ALS [8,9]. For that reason, an EMG is recommended to clarify the nature of the fasciculations. Even normal EMG requires that patients are followed up by a physician for a minimum of 4-5 years before a decision can be made about the benign nature of fasciculations [7].

Dysphagia

The characteristic of dysphagia is fundamental in understanding its possible causes. Dysphagia for solids is an alarm sign for a GI disease, so an EGD is recommended for such patients [10,11]. It is rarely the presenting symptom of gastroesophageal reflux disease (GERD); however, it is present in 30% of patients with reflux esophagitis. At the same time, patients can have severe esophagitis or Barret’s esophagus and be symptom-free and have no heartburn [11]. In this case, the most consistent complaint was terminal oropharyngeal dysphagia. However, the occasional cough for liquids was a confounding factor that did not let us rule out a neurological condition. The sinus CT was solicited to exclude an anatomical abnormality.

Differential diagnosis

MND

In one-third of patients with ALS, the bulbar muscles are the first to be affected, and the initial symptoms consist of dysarthria and dysphagia [5]. In this case, the lack of gastrointestinal symptoms, the presence of widespread fasciculations, and the onset of dysphagia beginning one month after reporting facial fasciculations raised red flags. However, factors against the diagnosis were the age of the patient, the absence of muscle wasting/weakness, and no abnormal findings on neurological examination. We did not consider this as the most likely scenario.

Stress and anxiety

After the first month of persistent fasciculations, the patient became fearful of the eventual onset of ALS, causing a considerable amount of stress, a common cause of fasciculations [2]. This was amplified when he started having dysphagia. Also, symptoms of anxiety have been associated with functional impairment of swallowing [12], making it possible that both dysphagia and fasciculations were manifestations of a constant state of anxiety. For this reason, this was the most accepted differential diagnosis.

Iatrogenic

Methylphenidate causes an increase in the synaptic concentration of dopamine and norepinephrine, acting as an indirect catecholaminergic agonist [13]. It is theorized that these hormones stimulate the prefrontal cortex, translating into more ability to ignore environmental distractions, planning, and motor inhibitory responses [14]. It is unclear what the exact mechanism is, but according to the European Medicines Agency (EMA), fasciculations are an uncommon side effect (<1%) of methylphenidate [15,16]. Although a rare and unlikely side effect, we considered it a possibility, but methylphenidate could not be the cause of the dysphagia.

Benign

This was supported by the absence of muscle weakness/wasting and abnormalities on neurological examination and due to the fact that over 1% of the population suffers from benign fasciculation [7]. However, similar to the iatrogenic differential, it provided no satisfactory explanation for dysphagia.

Two Different Explanations

As stated, the most plausible scenario that could explain the chronological order of events would be either MND with bulbar onset or anxiety-/distress-related syndrome. The rest of the differential diagnoses could only adequately explain one of the symptoms. For this reason, we found it reasonable to take into consideration that two different events/diseases were simultaneously happening, with an unfortunate chronological order.

Final diagnosis

The results of the complementary diagnostic tests demonstrated no pathological changes in the EMG, ruling out a possible motor neuron disease. At the same time, the upper digestive tract endoscopy (UDE) revealed a grade C esophagitis that provided an explanation for the dysphagia. This means that the dysphagia was not related to the fasciculations. Presented with this new reality, we had three options on the table that could adequately explain the presence of fasciculations: stress, iatrogenic, and benign fasciculation syndrome (BFS). To establish a BFS diagnosis, the patient needs to be monitored for 4-5 years, meaning that, for now, it was not possible to confirm this.

Dysphagia was resolved after two months of esomeprazole treatment, confirming this hypothesis. However, fasciculations persisted, leading us to consider an iatrogenic cause in detriment to anxiety. After stopping methylphenidate for one month, the patient reported a substantial improvement in fasciculations, confirming GERD and iatrogenic causes for dysphagia and fasciculations, respectively.

## Conclusions

This challenging clinical case strengthens the role of Physical Medicine and Rehabilitation as a diagnostic specialty. Causation does not necessarily mean correlation. In the reported case, the presenting symptoms were uncommon side effects of the respective causes, which made it more difficult to approach. This case highlights the importance of a holistic and, simultaneously, personalized approach to any patient.
